# Need for enforcement of ethicolegal education – an analysis of the survey of postgraduate clinical trainees

**DOI:** 10.1186/1472-6939-6-8

**Published:** 2005-08-06

**Authors:** Mayumi Mayeda, Kozo Takase

**Affiliations:** 1 Department of Health Science Policies Division of Research Development, Graduate School, Tokyo Medical and Dental University, 1-5-45 Yushima, Bunkyo-ku, Tokyo 113-8519, Japan

## Abstract

**Background:**

The number of medical lawsuits in Japan was between 14 and 21 each year before 1998, but increased to 24 to 35 per year after 1999. There were 210 lawsuits during this 10-year period. There is a need for skills and knowledge related to ethics, which is as fundamental to the practice of medicine as basic sciences or clinical skills. in Japan education in ethics is relatively rare and its importance is not yet recognized. Establishing ethics education using legal precedents, which has already been achieved in Western countries, will be a very important issue in Japan. In the present study, a questionnaire survey was conducted among graduate intern doctors, in order to investigate whether ethics education using precedents might have a positive effect in Japan.

**Methods:**

In 2002, a questionnaire survey entitled Physicians' Clinical Ethics was carried out in a compulsory orientation lecture given to trainees before they started clinical practice in our hospital. The attendees at this lecture were trainees who came from colleges in various districts of Japan. During the lecture, 102 questionnaires were distributed, completed by attendees and collected. The recovery rate was 100%. The questionnaire consisted of 22 questions (in three categories), of which 20 were answered by multiple choices, and the other two were answered by description. The time required to complete the questionnaire was about 10 minutes.

**Results:**

The recovered questionnaires were analyzed using statistical analysis software (SPSS for Windows, Release 10.07J-1/June/2000), in addition to simple statistical analysis. answers using multiple choices for the 20 questions in the questionnaire were input into SPSS. The principal component analysis was performed for each question. As a result, the item that came to the fore was "legal precedent". Since many intern doctors were interested in understanding laws and precedents, learning about ethical considerations through education using precedents might better meet with their needs and interests.

**Conclusion:**

We applied a new method in which the results of principal component analysis and frequencies of answers to other questions were combined. From this we deduced that the precedent education used in Western countries was useful to help doctors acquire ethical sensitivity and was not against their will. A relationship was found between reading precedents and the influence of lawsuits, and it was thought that student participation-type precedent education would be useful for doctors in order to acquire ethical sensitivity.

## Background

The conditions for acquiring a physician's license in Japan are considered to be less strict than those in other advanced countries [1]. Consequently, only technical aspects have been emphasized in medical education, and paternalistic treatment has continued in clinical practice [2]. However, as the incidence of patient requests to physicians has increased following establishment of the self-decision-making rights of patients [3], contradictions that cannot be addressed adequately by conventional medical education or clinical settings have arisen [4]. In medical education, the amount of information learned by students has been increasing year by year, in line with scientific advances. Clinical medicine is thought to be shifting toward a patient-oriented contract, and in this model, a patient's right to autonomy as expressed by the term "informed decision" [5], and a physician's right to exercise his/her professional discretion are two of the main concepts [6].

Therefore, the six-year study period for medical students is not adequate, and medical education that allows sufficient time for learning knowledge and techniques is needed. The importance of culture as a part of medical education has been raised [7] and physicians' ethical views have become an issue. There is a need for skills and knowledge related to ethics, which is as fundamental to the practice of medicine as basic sciences or clinical skills [8].

Given this situation, the Medical Education Model Core Curriculum – Educational Contents Guideline (Core Curriculum) [9] – was announced by the Ministry of Education, Culture, Sports, Science and Technology when the postgraduate clinical trainee system became obligatory (in the 2004 financial year). The core curriculum, which is a standardized medical education guideline presented by the Ministry of Education, Culture, Sports, Science and Technology in Japan, shows the basic principles of educational reform [10] and promotes the guidelines as an educational form that does not have the borders which usually exist in colleges between culture, basic medicine and clinical medicine [11]. In particular, it deserves special attention that "medical ethics" was first established as a general education subject in the core curriculum [12]. To change the conventional cramming education to problem solving-type education [13], the core curriculum is expected to act as a catalyst for each college in introducing ethics (including law and law cases) as an elective.

Against this background, there has recently been an increase in medical lawsuits in Japan. The number of medical lawsuits in Japan was between 14 and 21 each year before 1998, but increased to 24 to 35 per year after 1999. There were 210 lawsuits during this 10-year period [14]. Possible factors associated with this increase in lawsuits are loss of rapport between the physician and patient [15,16], neglect of the physician's duty to explain [17], manipulation of medical records [18] and secretive behavior [19]. As medical technology becomes more highly developed, minor mistakes by physicians may result in serious harm to patients [20]. In addition, consistent differences in malpractice experience exist among medical schools [21]. To reduce these lawsuits, acquiring a balance in training in both technique and ethics at the stage of medical education might be necessary [22].

As mentioned above, if ensuring that medical students acquire a sense of ethics results in a decrease in medical lawsuits, then ethics education for medical students using legal precedents as subject matter might be the best option. Courses in medical ethics are becoming an integral part of the curricula for many medical schools in Europe. However, in Japan education in ethics is relatively rare and its importance is not yet recognized. Establishing ethics education using legal precedents, which has already been achieved in Western countries, will be a very important issue in Japan.

In the present study, a questionnaire survey was conducted among graduate intern doctors, in order to investigate whether ethics education using precedents might have a positive effect in Japan.

The trainee period is a critical time for fostering ethical reasoning [23]. The survey of awareness among intern doctors during this period of transition from medical student to physician may provide key points for pre-graduate and postgraduate education. There have been no previous reports about questionnaire surveys of postgraduate clinical trainees in Japan. The present study was conducted in order to gain a better understanding of the ethical sensitivity of postgraduate clinical trainees in Japan and reflect obtained results in medical education.

## Methods

In 2002, a questionnaire survey entitled Physicians' Clinical Ethics was carried out in a compulsory orientation lecture given to trainees before they started clinical practice in our hospital (Appendix 1) [see Additional file 1]. "Clinical ethics" indicates "the personal (confidential) relations between doctors and patients", "clinical orders that doctors must carry out" and "morality to maintain these relations and orders". The attendees at this lecture were trainees who came from colleges in various districts of Japan. The study was approved by the Center for Postgraduate Education of Tokyo Medical and Dental University, in compliance with the internal regulations of the hospital.

During the lecture, 102 questionnaires were distributed, completed by attendees and collected. The recovery rate was 100%. The questionnaire consisted of 22 questions (in three categories), of which 20 were answered by multiple choices, and the other two were answered by description. The time required to complete the questionnaire was about 10 minutes. The recovered questionnaires were analyzed using statistical analysis software (SPSS for Windows, Release 10.07J-1/June/2000), in addition to simple statistical analysis. Correlation analysis was performed to evaluate the similarity among the items. In particular, principal component analysis was performed to find factors in the background of physicians' awareness. The principal component analysis was used to comprehend entire trends that could not be obtained through comparison of frequencies within each answer or through one-to-one correlation analysis. Only one component to indicate the entire trend was not calculated, but plural components were worked out in descending order of degrees to explain factors.

First, answers using multiple choices for the 20 questions in the questionnaire were input into SPSS. The principal component analysis was performed for each question. There were many questions that had various dispersed principal components and whose answering trend could not be explained. Interestingly, in respect of Question A ("Disclosure of medical record"), the entire trend could be explained by the top two extracted components. Consequently, the item that came to the fore was "legal precedent".

## Results

### Disclosure of medical records and law case education

As a first step, principal component analysis was performed as for the intern doctors' answers (multiple choice) to the question "Why do you think disclosure of medical records is required?"

**Figure 1:**
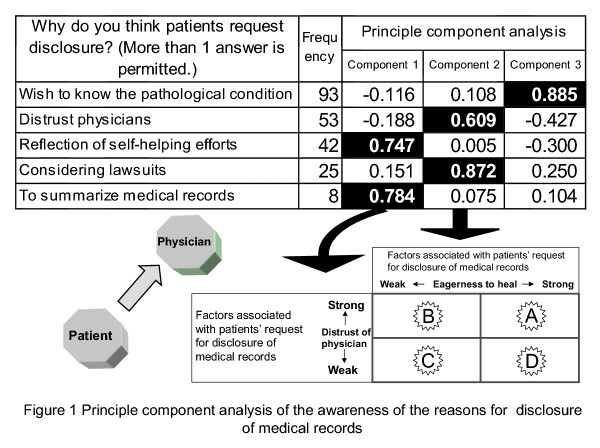
**Principle component analysis of the awareness of the reasons for disclosure of medical records**. Principal component analysis was performed as for the intern doctors' answers (multiple choice) to the question "Why do you think disclosure of medical records is required?" To evaluate the relationship between components 1 and 2, they were placed in a 2 × 2 matrix, with attention paid to each component's strengths and weaknesses, and classified into (A), (B), (C) and (D).

The characteristics of the three components extracted from the answers were elicited in reference to question items. Component 1 was one of the top two components (with a cumulative variance of 49%) and was considered to reflect "eagerness to heal" because the two most frequent choices were "reflect self-helping efforts" and "to summarize medical records". Component 2 may reflect "distrust of physicians" because the frequencies of "distrust physicians" and "considering lawsuits" were high (see Figure 1).

**Figure 2:**
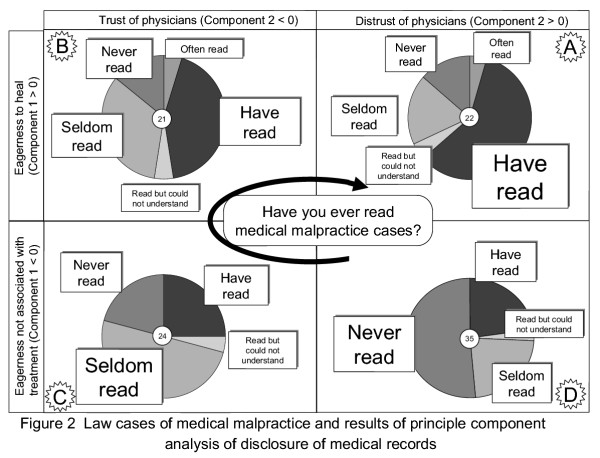
**Law cases of medical malpractice and results of principle component analysis of disclosure of medical records**. A particularly strong trend could be observed in relation to answers (A), (B), (C) and (D) with respect to the answers to the question "Have you read literature concerning medical malpractice precedents?" Answers were: (A) in 22 subjects, (B) in 21, (C) in 24, and (D) in 35. In (A) to (D), the percentages of answers to the above question were expressed as pie graphs.

In addition, to evaluate the relationship between components 1 and 2, they were placed in a 2 × 2 matrix, with attention paid to each component's strengths and weaknesses, and classified into (A), (B), (C) and (D) (see Figure 2). (A) indicated that "the disclosure was required since patients themselves had willingness to be cured or patients had distrust for doctors"; (B) indicated that "the disclosure was required only for patient's willingness to be cured"; (C) indicated that "the disclosure was required due to simple interests"; and (D) indicated that "the disclosure was required since patients had distrust for doctors".

The following analysis was performed by combining the above results for (A), (B), (C) and (D) with answers to other questions. As a result, a particularly strong trend could be observed in relation to answers (A), (B), (C) and (D) with respect to the answers to the question "Have you read literature concerning medical malpractice precedents?" Answers were: (A) in 22 subjects, (B) in 21, (C) in 24, and (D) in 35. In (A) to (D), the percentages of answers to the above question were expressed as pie graphs. As a result, the interesting fact emerged that doctors who selected (D) (followed by (C), (B) and (A)) had more experience of reading legal precedents. This indicated that there was some relation between interns' reading legal precedents and their reason given for "Why do patients require the disclosure of medical records?" An arrow directed from "patients" to "doctors" in Figure 1 shows how doctors perceive patients' evaluations of doctors.

### Patients' awareness

Subsequently, which answer ((A), (B), (C) or (D)) best reflects the thoughts of patients was investigated with reference to the reasons actually given by patients for requesting medical records disclosure. At 3-year intervals, The Japanese Ministry of Health, Labour, and Welfare produces a "patients' awareness survey", based on the contents/results of a study of treatment-receiving behavior.

**Figure 3:**
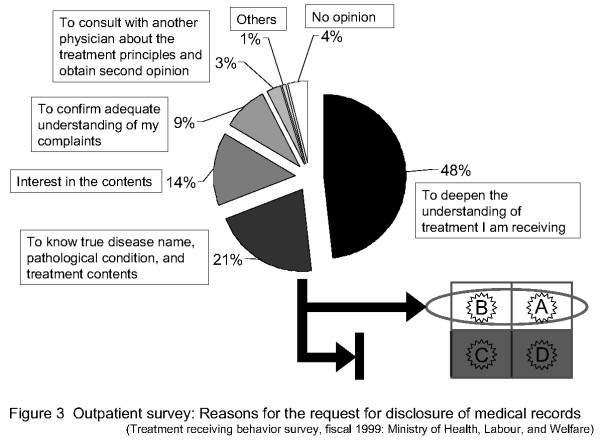
**Outpatient survey: Reasons for the request for disclosure of medical records (Treatment receiving behavior survey, fiscal 1999: Ministry of Health, Labour, and Welfare)**. With respect to the reason that outpatients wish to know the contents of their medical records, about 50% of patients selected "to deepen the understanding of treatment I receive". The second highest percentage of patients selected "to know the true disease name, disease condition, and treatment contents". Thus, patients' "eagerness to heal" was the reason given by most patients.

I compared the 1999 version of the ministry's survey with "eagerness to heal" in this questionnaire survey (see Figure 3) [24]. With respect to the reason that outpatients wish to know the contents of their medical records, about 50% of patients selected "to deepen the understanding of treatment I receive". The second highest percentage of patients selected "to know the true disease name, disease condition, and treatment contents". Thus, patients' "eagerness to heal" was the reason given by most patients. Based on these results, the reasons given by patients differed from those given by doctors who selected (C) and (D) (i.e., the disclosure was required due to simple interests or because patients had distrust for doctors), and was close to those of doctors who selected (A) and (B) (i.e., the disclosure was required for patients' willingness to be cured). Therefore, in terms of mutual understanding between doctors and patients, answers (A) and (B) were preferable to (C) and (D).

### Lawsuits and eagerness to study

**Figure 4:**
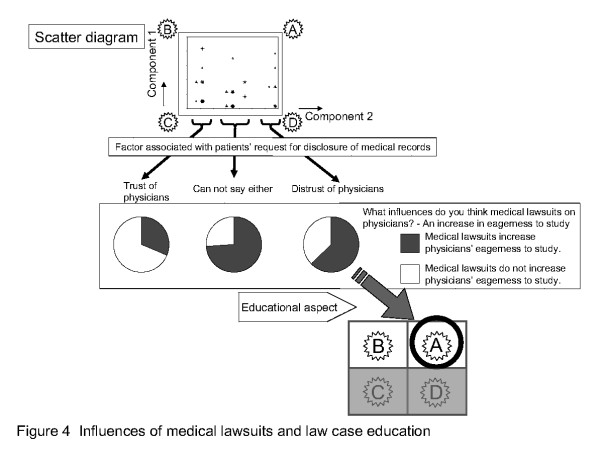
**Influences of medical lawsuits and law case education**. Answers to the question "What influences do you think medical lawsuits have on physicians? The percentage of "do not increase eagerness to study" responses was higher for "trust of physicians", and that of "do increase eagerness to study" was higher for "undecided" and "distrust of physicians". Therefore, answer (A) was more desirable than (B) in terms of to "increase eagerness to study".

Subsequently, on the assumption that precedent education (i.e., ethical education based on precedents) was useful to educate intern doctors, we investigated which of answers (A) and (B) was preferable. Answers to the question "What influences do you think medical lawsuits have on physicians? [- An increase in eagerness to study]" were used (see Figure 4). Respondents had the following two choices: Medical lawsuits "do not increase physicians' eagerness to study" and "do increase physician's eagerness to study". These answers were combined with Figure 1 and further classified according to "trust or distrust of physicians" with cluster analysis.

In cluster analysis, common points are extracted and grouped. As shown in the scatter diagram of factor scores (see Figure 4) for disclosure of medical records, the following three definite clusters emerged: "trust of physicians", "undecided", and "distrust of physicians". The percentage of "do not increase eagerness to study" responses was higher for "trust of physicians", and that of "do increase eagerness to study" was higher for "undecided" and "distrust of physicians". Therefore, answer (A) was more desirable than (B) in terms of to "increase eagerness to study". (A) can be defined as the following balanced state: as physicians read more cases, they increase their awareness of patients' eagerness to heal and distrust of physicians, and increase their eagerness to study and aim at reliable medical treatment to eliminate this distrust. Thus, law case education for trainees has favorable effects, and its incorporation into medical education should be useful.

### Effects of law case education

**Figure 5:**
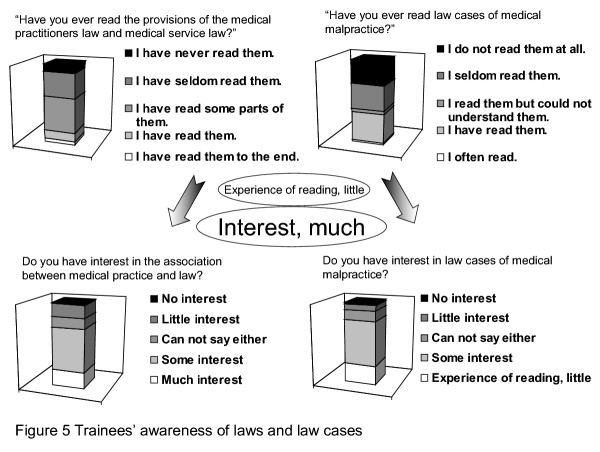
**Trainees' awareness of laws and law cases**. The general effects of law case education were evaluated (see Figure 5). The survey showed that only a low percentage of the trainees had ever read "provisions of the medical practitioners law/medical service law" and "law cases of medical malpractice" in their entirety and with complete understanding.

The general effects of law case education were evaluated (see Figure 5). The survey showed that only a low percentage of the trainees had ever read "provisions of the medical practitioners law/medical service law" and "law cases of medical malpractice" in their entirety and with complete understanding. This may represent trainees' perceptions that reading law cases is difficult due to complex technical terms, long sentences, and obscure expressions. However, many trainees had some interest in "medical practice and laws" and "law cases of medical malpractice". Therefore, high-level law and law case education may not always be necessary for trainees who rarely read laws or cases. Since many intern doctors were interested in understanding laws and precedents, learning about ethical considerations through education using precedents might better meet with their needs and interests.

### Law case education and the influences of medical lawsuits

**Figure 6:**
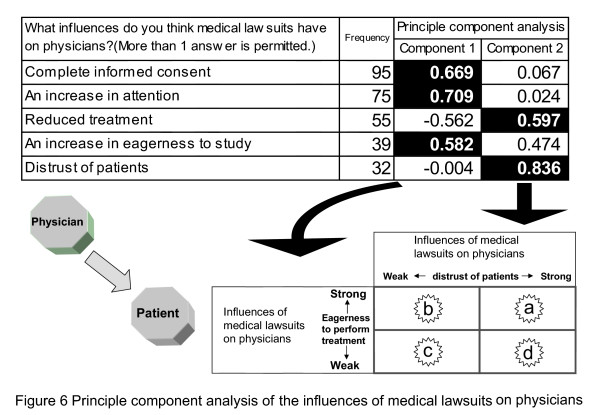
**Principle component analysis of the influences of medical lawsuits on physicians**. Principal component analysis of trainee's answers to the question "What influences do you think medical lawsuits have on physicians?" was performed. As a result of principal component analysis, two components were extracted. Component 1 was defined as "eagerness to perform treatment" and Component 2 as "distrust of patients".

We next considered what matters would be appropriate for precedent education. The purpose of precedent education is to strengthen the ethical sensitivity of doctors toward patients. As a first step, principal component analysis of trainee's answers to the question "What influences do you think medical lawsuits have on physicians?" was performed (see Figure 6). The analysis method was the same as that shown in Figure 1. However, since the subject was the attitude of doctors toward patients, an arrow is directed from "doctors" to "patients".

**Figure 7:**
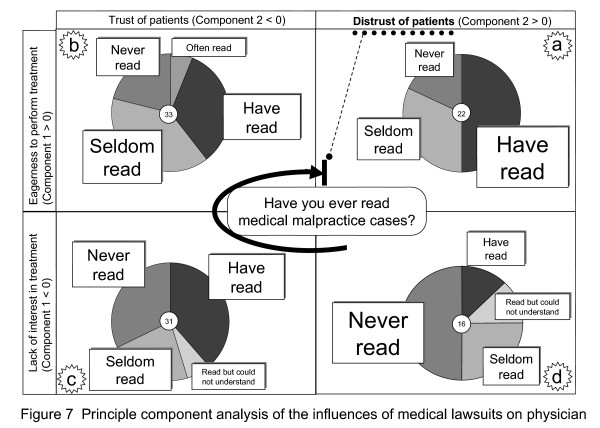
**Principle component analysis of the influences of medical lawsuits on physician**. When (a) to (d) were combined with the answers to the question "Have you ever read law cases of medical malpractice?", experience in reading cases increased in the order of (d), (c), (b), and (a). The results of the survey indicated that doctors who had read more precedents showed more distrust toward patients.

As a result of principal component analysis, two components were extracted. Component 1 was defined as "eagerness to perform treatment" and Component 2 as "distrust of patients". To evaluate the relationship between components 1 and 2, they were classified according to their strength into the following four items: (a) eagerness to perform treatment and distrust of patients; (b) eagerness to perform treatment and trust of patients; (c) lack of interest in treatment but trust of patients; and (d) lack of interest in treatment and distrust of patients. When (a) to (d) were combined with the answers to the question "Have you ever read law cases of medical malpractice?", experience in reading cases increased in the order of (d), (c), (b), and (a) (see Figure 7). The results of the survey indicated that doctors who had read more precedents showed more distrust toward patients.

## Discussion

We applied a new method in which the results of principal component analysis and frequencies of answers to other questions were combined. From this we deduced that the precedent education used in Western countries was useful to help doctors acquire ethical sensitivity and was not against their will. However, since precedent education could increase doctors' distrust toward patients, it might be necessary to be selective about the subjects of precedent education. (Figures 2 and 7 seem to indicate the same results at the first glance. However, doctors' awareness of patients' distrust toward doctors and doctors' aggravating distrust toward patients greatly differed in quality.)

If Western-style, precedent-based education, is introduced in Japan, the issues listed below need to be addressed, while Japanese precedents might also be helpful when considering educational content.

Complex and obscure expressions and technical terms are used in law cases in Japan. People (other than those who have a law education) cannot read and understand the cases' provisions or issues. This suggests that simply reading law in medical education is a problem. Student participation-type education, in which students learn not by simply reading and remembering the precedents and articles, but by comprehending particular cases, would be a useful way to introduce precedent education into medical education. We also believe that teaching using a Socratic method based on notable medical malpractice cases would be appropriate.

In Japan, paternalistic-like treatment continues to occur in clinical practice. It could be a relatively simple process to help doctors to understand the mental pain that patients experience and their distrust toward doctors, by carefully teaching them the processes of medical malpractice suits using past precedents. This might lead doctors to develop a deeper understanding of patients' standpoints.

Cases about accountability violation, which becomes problematic in many malpractice suits in Japan, might be suitable subject matter for education using precedents. For instance, in 2001, the Supreme Court decided (in a case about mammary amputation) that doctors have a responsibility to disclose even a treatment that has not yet been technically established in order to achieve accountability [25]. In the case, a patient with breast cancer had pleaded with a doctor not to remove the breast, and to perform breast conservation therapy. However, the doctor removed the entire breast and was found to have been derelict in accountability. In education using precedents it will be necessary for students to learn the importance of accountability.

Many patients feel dissatisfied with the treatment they receive from doctors. The facts and issues shown in precedent cases reflect this distrust of patients toward doctors, and can increase the distrust that doctors feel toward patients if they are read without appropriate instructions. Doctors can have deep distrust toward patients (see Figure 7a) even when patients have a strong willingness to be cured. Education needs to be directed at helping doctors gain the trust of patients (see Figure 2A) while achieving a balance between doctors' willingness to cure and their trust toward patients (see Figure 7b). That is, the education must increase the mutual trust between doctors and patients [26,27]. Illustrating the standpoints of each party, through the facts and issues shown in the precedents, would be the first step toward developing the trust that doctors feel toward patients.

Japanese students have difficulty with student participation-type lessons. To change the conventional cramming-type education to a more problem solving-type education is not contrary to trainees' wishes, based on the results of the questionnaire, and does not require high-level knowledge of law cases.

Another possible subject matter could be precedents about medical record alteration and concealment, which are often seen in Japan. In 2001, there was a case in which the medical records of a patient who died due to malpractice during an operation at the University Hospital (the case involved Tokyo Women's Medical University [28]) were altered. The alteration was systematically organized by the entire medical team, and the case had a significant impact on society. When an error is made in medical practice in Japan, the evidential facts tend to be hidden. It is necessary for students to learn that understanding the patient's viewpoint and adopting an honest attitude to malpractice would be in their own best interests.

Student participation-type education (i.e., education involving simulation, problem-based learning [29,30], role-playing [31] and skills laboratories) is rarely used in Japan. An education method for law and ethics in which a small group of intern doctors and teachers of ethics (who can play an important role in modeling the very nature of ethics) [32], maintain smooth communication and foster mutual understanding from the standpoints of doctors and patients, corresponds to the traditional custom of Asian countries including Japan, where ethical sensitivity is established in families and communities. This method might be a highly effective way for doctors to acquire ethical sensitivity.

Lectures in which "mock trials" are performed and discussed, with students playing the parts of the accuser, accused and judge, should be useful. The jury system has not yet been introduced in Japan, although it will be in the near future (although approximately 70% of citizens do not want to be part of a jury). Ideally, any simulated trials should incorporate juries.

## Conclusion

The analysis was performed following an increasing incidence of medical lawsuits in Japan. The results of our survey indicated that precedent education performed in Western countries would also be useful in Japan, and would not go against the attitude of doctors toward precedents and ethics. The relationship between precedents and ethics came to the forefront in a questionnaire survey of graduate intern doctors, which had not previously been conducted in Japan, and the principal component analysis developed from a new viewpoint with [a narrower focus]. A relationship was found between reading precedents and the influence of lawsuits, and it was thought that student participation-type precedent education would be useful for doctors in order to acquire ethical sensitivity. From this study, certain educational devices using materials and reminders based on Japanese precedents could be proposed. These devices might be necessary when introducing precedent-based education in Japan.

## Competing interests

The author(s) declare that they have no competing interests.

## Authors' contributions

MM: Whole of the study and writing the manuscript.

KT: Review of questionnaire plan and manuscript.

## Pre-publication history

The pre-publication history for this paper can be accessed here:



## Supplementary Material

Additional file 1 Questionnaire of physicians' clinical ethics Click here for file
