# Supplementary material for: Need for enforcement of ethicolegal education – an analysis of the survey of postgraduate clinical trainees
Source: BMC Med Ethics. 2005 Aug 6;6:8. doi: 10.1186/1472-6939-6-8 (PMC1192799; doi:10.1186/1472-6939-6-8)
Supplement: Additional file 1 — Questionnaire of physicians' clinical ethics [file 1472-6939-6-8-S1.doc]

[Additional file 1]

**Questionnaire on physicians’ clinical ethics**

The environment surrounding medicine is changing on a daily basis. Doctors should consider clinical ethics in their relationships with patients. “Clinical ethics” indicates “the personal (confidential) relations between doctors and patients”, “clinical orders that doctors must carry out” and “morality to maintain these relations and orders”. It is believed that clinical ethics has an influence on medical services in general. Please answer the following questions with the physician’s relations with patients in mind. Your cooperation is greatly appreciated.

Circle applicable answers*.*

1. **Do you think that rapport can be established between a physician and patient in medical practice?**

1. Very applicable 2. Generally applicable 3. Undecided 4. Slightly applicable 5. Not applicable 6. Others

1. **In your personal opinion, is disclosure of medical reports a good idea?**

1. Very applicable 2. Generally applicable 3. Undecided 4. Slightly applicable 5. Not applicable 6. Others

1. **Do you think it is good that patients obtain knowledge about their own disorders through the Internet and books?**

1. Very applicable 2. Generally applicable 3. Undecided 4. Slightly applicable 5. Not applicable 6. Others

(3**)-1 Why do you think so?** ＿＿＿＿＿＿＿＿＿＿＿＿＿＿＿＿＿＿＿＿

1. **Do you think that palliative care is good?**

1. Very applicable 2. Generally applicable 3. Undecided 4. Slightly applicable 5. Not applicable 6. Others

1. **There are some overseas countries with established euthanasia laws. Do you think that euthanasia is good?**

1. Very applicable 2. Generally applicable 3. Undecided 4. Slightly applicable 5. Not applicable 6. Others

1. **Similarly, do you think that death with dignity is good?**

1. Very applicable 2. Generally applicable 3. Undecided 4. Slightly applicable 5. Not applicable 6. Others

1. **Do you think that the second opinion system is good?**

1. Very applicable 2. Generally applicable 3. Undecided 4. Slightly applicable 5. Not applicable 6. Others

1. **Do you think that medical treatment that relies heavily on drugs is good?**

1. Very applicable 2. Generally applicable 3. Undecided 4. Slightly applicable 5. Not applicable 6. Others

1. **Do you think that drug administration affects the mental state of patients?**

1. Very applicable 2. Generally applicable 3. Undecided 4. Slightly applicable 5. Not applicable 6. Others

(10) **Do you think that you understand the “clinical trial” system?**

1. Very applicable 2. Generally applicable 3. Undecided 4. Slightly applicable 5. Not applicable 6. Others

(11**) Do you think that patients understand the “clinical trial” system?**

1. Very applicable 2. Generally applicable 3. Undecided 4. Slightly applicable 5. Not applicable 6. Others

(12) **Are you interested in the association between medical practice and law?**

1. Very applicable 2. Generally applicable 3. Undecided 4. Slightly applicable 5. Not applicable 6. Others

(13) **Do you think that there is a phenomenon of reduced medical practice due to legal issues?**

1. Very applicable 2. Generally applicable 3. Undecided 4. Slightly applicable 5. Not applicable 6. Others

(14) **Have you ever read law cases about medical malpractice?**

1. Very applicable 2. Generally applicable 3. Undecided 4. Slightly applicable 5. Not applicable 6. Others

(15) **Are you interested in law cases about medical malpractice?**

1. Very applicable 2. Generally applicable 3. Undecided 4. Slightly applicable 5. Not applicable 6. Others

(16) **You may often hear the word “ethics”. Do you think that you understand “clinical ethics”?**

1. Very applicable 2. Generally applicable 3. Undecided 4. Slightly applicable 5. Not applicable 6. Others

(17) **Have you ever read the provisions of the medical practitioners’ law and medical service law?**

1. Very applicable 2. Generally applicable 3. Undecided 4. Slightly applicable 5. Not applicable 6. Others

A: **Why do you think patients request disclosure of medical records? Circle all applicable answers.**

1. It is a reflection of self-help efforts 2. Because they distrust physicians

3. To summarize medical records 4. Because they are considering medical lawsuits 5. They wish to know their pathological condition

6. Others_______.

B. **What influences do you think medical lawsuits have on physicians? Circle all applicable items.**

1. Reduced treatment 2. Distrust of patients 3. An increase in attention

4. An increase in eagerness to study

5. Complete informed consent of patients 6. Others_________

C**. When drugs are prescribed, to what extent do you think physicians should explain? Circle all applicable items.**

1. Names of drugs 2. Methods of drug use 3. Effects of drugs

4. Side effects of drugs 5. Matters requiring attention

6. Others _________

D. **Describe your understanding of the term “clinical ethics”.** ______________

Thank you.
